# *Plasmodium falciparum* Merozoite Surface Proteins Polymorphisms and Treatment Outcomes among Patients with Uncomplicated Malaria in Mwanza, Tanzania

**DOI:** 10.1155/2022/5089143

**Published:** 2022-11-23

**Authors:** Karol J. Marwa, Eric Lyimo, Eveline T. Konje, Anthony Kapesa, Erasmus Kamugisha, Göte Swedberg

**Affiliations:** ^1^Department of Pharmacology, Catholic University of Health and Allied Sciences, Mwanza, Tanzania; ^2^National Institute for Medical Research, Mwanza, Tanzania; ^3^Department of Community Medicine, Catholic University of Health and Allied Sciences, Mwanza, Tanzania; ^4^Department of Biochemistry, Catholic University of Health and Allied Sciences, Mwanza, Tanzania; ^5^Department of Medical Biochemistry and Microbiology, Uppsala University, Uppsala, Sweden

## Abstract

**Background:**

The severity of malaria infection depends on the host, parasite and environmental factors. Merozoite surface protein (msp) diversity determines transmission dynamics, *P. falciparum* immunity evasion, and pathogenesis or virulence. There is limited updated information on *P. falciparum* msp polymorphisms and their impact on artemether-lumefantrine treatment outcomes in Tanzania. Therefore, this study is aimed at examining msp genetic diversity and multiplicity of infection (MOI) among *P. falciparum* malaria patients. The influence of MOI on peripheral parasite clearance and adequate clinical and parasitological response (ACPR) was also assessed.

**Methods:**

Parasite DNA was extracted from dried blood spots according to the manufacture's protocol. Primary and nested PCR were performed. The PCR products for both the block 2 region of msp1 and the block 3 regions of msp2 genes and their specific allelic families were visualized on a 2.5% agarose gel.

**Results:**

The majority of the isolates, 58/102 (58.8%) for msp1 and 69/115 (60.1%) for msp2, harboured more than one parasite genotypes. For the msp1 gene, K1 was the predominant allele observed (75.64%), whereas RO33 occurred at the lowest frequency (43.6%). For the msp2 gene, the 3D7 allele was observed at a higher frequency (81.7%) than the FC27 allele (76.9%). The MOIs were 2.44 for msp1 and 2.27 for msp2 (*p* = 0.669). A significant correlation between age and multiplicity of infection (MOI) for msp1 or MOI for msp2 was not established in this study (*rho* = 0.074, *p* = 0.521 and *rho* = −0.129, *p* = 0.261, respectively). Similarly, there was no positive correlation between parasite density at day 1 and MOI for both msp1 (*rho* = 0.113, *p* = 0.244) and msp2 (*rho* = 0.043, *p* = 0.712). The association between MOI and ACPR was not observed for either msp1 or mps2 (*p* = 0.776 and 0.296, respectively).

**Conclusions:**

This study reports high polyclonal infections, MOI and allelic frequencies for both msp1 and msp2. There was a lack of correlation between MOI and ACPR. However, a borderline significant correlation was observed between day 2 parasitaemia and MOI.

## 1. Introduction

Sub-Saharan Africa has the highest burden of *Plasmodium falciparum* malaria, with eleven countries accounting for 70% of all cases and 94% of the recorded deaths [1]. Malaria still remains a public health challenge in Tanzania despite the reported decrease in prevalence of about 10% over the past 10 years. The severity of malaria infection depends on the host, parasite and environmental factors [2]. Parasite factors such as merozoite surface protein polymorphism (msp), *Plasmodium falciparum* surface protein (Pfs47), and apical membrane antigen (AMA1) may influence treatment outcomes.

Merozoite surface protein diversity determines transmission dynamics, *P. falciparum* immunity, evasion and virulence. The merozoite surface proteins are regarded as a potential target for malaria vaccine development [3, 4] and antigenic polymorphisms have been associated with reduced vaccine efficacy [5, 6].

Diversity in merozoite surface protein (msp) genes is also employed in the characterization of *P. falciparum* strains. Among the blood stage surface antigens, merozoite surface protein 1 (msp1) and merozoite surface protein 2 (msp2) are the most commonly used markers for the identification of genetically distinct *P. falciparum* parasite populations [7]. The msp1 gene, located on chromosome 9 [8] is a major *P. falciparum* surface protein encoding a 185–215 kDa protein which is cleaved into several polypeptides during merozoite maturation and red cell invasion [7]. The msp1 gene may be divided into 17 blocks of diverse sequences flanked by conserved regions. Block 2 (a region near the *N*-terminal of the gene) is the most polymorphic part of the msp1 gene [9] and is grouped into three major allelic families, namely MAD20, K1 and RO33 based on the variable nucleotide sequence and copy number of repeats of block 2 [8].

The msp2 gene is located on chromosome 2 and is encoding the merozoite surface protein 2 which is a glycoprotein with an approximately 30 kDa [10, 11]. It is composed of five blocks whereby the central block (block 3) is the most polymorphic. The msp2 alleles are grouped into two allelic families, namely, FC27 and 3D7/IC1 [12, 13]. Fragment size polymorphisms in MAD20, K1 and RO33 (for msp1) and FC27 and 3D7 (for msp2) are used as molecular markers in studying *P. falciparum* malaria transmission dynamics and virulence [14]. Genotyping of msp1 and msp2 is also employed in differentiating recrudescence from reinfection in antimalarial efficacy studies.

Multiplicity of infection (MOI), also referred to as complexity of infection (COI), is defined as the average number of distinct parasite genotypes concurrently infecting a patient [15] or the number of different *P. falciparum* strains coinfecting a single host [16]. MOI is used as an indicator for malaria transmission and immune status (in areas with stable malaria transmission) [16]. Parasite genotype and MOI are suggested to modulate infection outcomes and are determined by recombination during the sexual life cycle and injection of multiple clones during mosquito bite, respectively [17].

Individuals in areas of high transmission experience multiple mosquito bites associated with multiple clones per bite, unlike in low transmission areas where most of the mosquito bites are associated with monoclonal strains [3, 18]. The low genetic diversity observed in low transmission areas is associated with a strong linkage disequilibrium (LD) and a defined structure of parasite populations, unlike in high transmission areas where there is a weak LD and nondefined population structures [19, 20]. Recent studies in Kenya and Myanmar reported no change in *P. falciparum* diversity and population structure after many years of intensive use of insecticide treated nets (ITNs) and insecticide residual spraying (IRS) despite a decline in malaria transmission due to the above interventions [19, 21]. In general, the mechanisms controlling parasite genetic diversity are many and complex [18].

The frequencies of the msp1 and msp2 alleles have been extensively reported globally. However, the influence of msp polymorphisms and MOI on *P. falciparum* treatment outcomes has been reported in very few areas [22] with contradicting findings. Studying MOI and the frequency of multiclonal infections is important in understanding malaria transmission intensity [15] in order to enable the establishment or modification of malaria control strategies. It is also imperative to determine the association between the msp polymorphisms and treatment outcomes. There is limited updated information on *P. falciparum* msp polymorphisms and their impact on transmission and treatment outcomes in Tanzania. Therefore, this study aimed at examining msp genetic diversity and MOI in a meso-endemic region and their association with peripheral parasite clearance and adequate clinical and parasitological response (ACPR) among *P. falciparum* malaria patients treated with artemether-lumefantrine.

## 2. Materials and Methods

### 2.1. Study Area and Patient Recruitment

This study was carried out at Karume Health Centre in Igombe, a semiurban and malaria meso-endemic area in Ilemela District, Mwanza region. The samples were collected during the rainy season of the year. Data for the study were prospectively collected from patients with uncomplicated *Plasmodium falciparum* (confirmed on the malaria rapid diagnostic test (MRDT)) malaria as part of the efficacy study involving artemether-lumefantrine and dihydro-artemisinin piperaquine published earlier [23]. Only patients on artemether-lumefantrine treatment were studied for parasite genetic diversity. The inclusion and exclusion criteria are described in the previous study [23]. Details on the patient's clinical examination and drug administration were in accordance with the Tanzania guideline for the management of malaria.

### 2.2. Follow-Up and Sample Collection

Patients were requested to return to the clinic for follow-up on days 1, 2, 3, 7, 14, 21, 28 and 35. Blood from finger pricks was collected on filter paper (FTA®Whatman paper) during the visits. The filter papers were then dried at room temperature and kept in sealed plastic bags. Thick and thin blood smears were stained by Giemsa (on each day of the visit) according to the standard protocol [24]. Parasite identification and counting were done by two independent experienced microscopists.

### 2.3. DNA Extraction and Genotyping for msp1, msp2, and Allelic Families

Parasite DNA was extracted from the dried blood spots (DBS) using the Life Sciences genomic DNA kit for dried spots according to the manufacture's protocol. Primers for genotyping of the block 2 for msp1, block 3 for msp2, specific allelic families for msp1 (MAD20, K1, and R033) and specific allelic families for msp2 (FC27 and 3D7) were in accordance to Somé et al. [25]. Primary and nested PCR was done using the method described previously [25]. The PCR products for both the msp1 and msp2 and their specific allelic families were visualized on a 2.5% agarose gel (containing red safe dye) under a UV illuminator. The band sizes were recorded. Recrudescence and reinfection were distinguished according to the WHO guideline [26].

### 2.4. Treatment Outcomes

The WHO 2015 protocol [27] was used to classify treatment outcomes at day 28 as early treatment failure (ETF), late clinical failure (LCF), late parasitological failure (LPF), and adequate clinical and parasitological response (ACPR). Treatment failures were also classified as recrudescence or reinfection after PCR correction.

### 2.5. Statistical Analysis

Statistical analyses were performed using STATA version 13.1 (Texas, USA). MOI was defined as the number of distinct parasite genotypes coexisting within a single infection [10] and was calculated as the maximum number of PCR fragments for block 2 (msp1) and 3 (msp2) regions visualized for each sample [22]. The mean MOI was estimated by dividing the total number of distinct msp1 or msp2 genotypes detected by the number of positive samples for the same markers [28]. The percentage of polyclonal infections in the study samples was computed based on the proportion of isolates with multiple genotypes per marker. Categorical data were compared using the chi-square test or Fisher exact test. Spearman's rank correlation coefficient was used to find out the relatedness of continuous variables. *p* value less than 0.05 was considered statistically significant.

## 3. Results

### 3.1. Allelic Diversity of *P. falciparum* msp1 and msp2 and MOI among Patients with Uncomplicated Malaria

**Figure 1:**
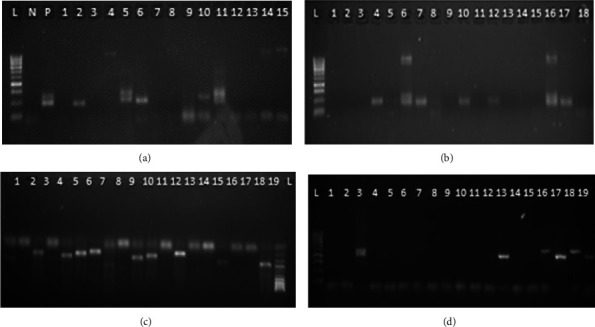
Gel image representing MAD20 (a), RO33 (b), 3D7 (c), and FC27 (d).

Alleles of msp1 and msp2 were classified according to the PCR amplified fragments. K1 was the predominant allele observed for msp1 (75.6%) and yielded 9 fragments (160–300 bp) as shown in Figure 1 and Table 1. The msp1 allele with the lowest frequency was RO33 (43.6%) and yielded 4 fragments (120–200 bp) as shown in Figure 1 and Table 1. For msp2, the 3D7 allele was observed at a higher frequency (81.7%) than FC27 (76.9%). The number and sizes of fragments are shown in Figure 1 and Table 1.

High MOI (above 2) was observed for both msp1 and msp2, but the difference between the two groups was not statistically significant (Table 2). The majority of the patients harboured polyclonal infections for both merozoite surface proteins although the difference between the two groups was not statistically significant. The overall mean MOI (msp1 and msp2 combined) was also high (2.36) (Table 2).

### 3.2. Association/Correlation between Age, Parasite Density, and ACPR

**Figure 2:**
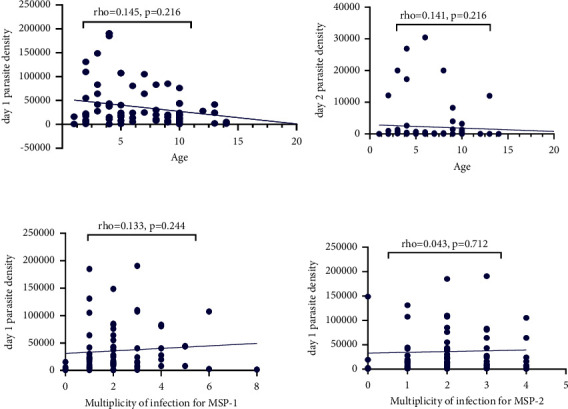
Spearman correlation between parasite density and age or multiplicity of infection.

A Spearman's correlation was run to assess the relationship between age and parasite density (at days 1 and 2). There was a negative correlation between age and parasite density at days 1 and 2, but this was not statistically significant (*rho* = 0.1415, *p*=0.2164 and *rho* = 0.1415, *p*=0.2164, respectively) (Figure 2). Parasite density at day 1 correlated negatively with ACPR, but the effect was not statistically significant (*rho* = −0.312, *p*=0.0054). However, there was a strong negative correlation between parasite density at day 2 and ACPR (*rho* = −0.4591, *p* < 0.001).

Significant correlation between age and MOI for msp1 or MOI for msp2 was not established in this study (*rho* = 0.074, *p* = 0.521 and *rho* = −0.1289, *p* = 0.261, respectively) as shown in Table 3. In addition, there was no positive correlation between parasite density at day 1 and MOI of msp1 (*rho* = 0.133, *p* = 0.244) (Table 3 and Figure 2). There was no strong positive correlation between parasite density at day 2 and MOI for msp1 (*rho* = 0.219, *p* = 0.054) (Table 3 and Figure 2).

For msp2, there was no strong positive correlation between parasite density at day 1 and MOI, for which there was a statistical significance (*rho* = 0.043, *p* = 0.712) (Table 3). There was also no strong positive correlation between parasite density at day 2 and the MOI for msp2, which was not statistically significant (*rho* = 0.006, *p* = 0.957) (Table 3). The association between MOI and ACPR was not observed for both msp1 and 2 (chi^2^ = 4.0361, *p* = 0.776 and chi^2^ = 4.9152, *p* = 0.296, respectively).

## 4. Discussion

The genetic diversity of *P. falciparum* is essential for the parasite to adapt to environmental changes, escaping host immunity through antigenic variation and develop resistance [29, 30]. The levels of parasite allelic diversity, outcrossing and gene flow have been reported to be highest in African populations when compared to South American and Southeast Asian populations [20, 31]. Understanding the genetic diversity and population structure of *P. falciparum* is essential for monitoring and evaluating malaria control strategies and interventions. The present study focused on the allelic diversity of *P. falciparum* and its influence on treatment outcomes utilizing isolates from patients with uncomplicated malaria treated with artemether-lumefantrine.

The predominant alleles were K1 for the msp1 gene and 3D7 for msp2 the gene. These findings are similar to those in other parts of the world [28, 32, 33]. This observation was not in conformity with previous studies done in Nigeria, Myanmar and Pakistan with regard to msp1, where by the predominant allele was MAD20 [7, 34, 35]. The lack of conformity was also observed for the msp2 gene in Nigeria and Sudan, with the predominant allele being FC27 [7, 11]. Host immune responses, changing environments and drug pressure may account for the inconsistencies in genetic diversity observed [36, 37]. Our findings suggest that K1 and 3D7 parasite strains are the common genotypes circulating in the study region. A similar observation was recorded in another area of the country (for the msp2 gene) [18], Nigeria and Senegal [38, 39].

*Plasmodium falciparum* infected malaria patients had high frequencies of polyclonal infection for both msp1 and msp2. The observed high polyclonal infection is not surprising in a malaria endemic and even meso-endemic area where it is common that patients will be infected by more than one distinct parasite genotype [15]. Multiclonal infections could be explained ecologically as a result of cotransmission of different parasite variants (coinfection) or superinfection [40, 41]. The high frequency of multiclonal isolates for both msp genes is alarming since multiclonal infections are predicted to be more virulent than single clone infections [15, 40] and are likely to be favoured by natural selection; thus, these infections are likely to be dominant in the population [42]. The *P. falciparum* mixed clone infections in humans also lead to cross-fertilization and recombination between parasite genomes in the mosquito vector [43]. These can lead to the selection of more virulent and competent parasites, endangering the effectiveness of the currently used ACTs.

The mean MOI for both msp1 and msp2 was high (between 2 and 2.5) at the study site, similar to findings from Sudan and Uganda [33, 44]. These values suggest the existence of a high malaria transmission rate. However, these MOI results were comparably lower than those documented more than ten years ago in other areas of the country, namely, Kilombero, Muheza and Ifakara [45, 46]. The difference in the observed MOI among patients with uncomplicated malaria over the past decade could be attributed to the differences in transmission intensity between the study sites as a result of the scaling up of malaria control and prevention strategies over the years. The differences in vector populations and human host immunity between the study sites and their changes with time could also account for the observed discrepancy.

The correlation between MOI with age has been reported with conflicting results; some studies have reported an inverse association between age and MOI, showing lower MOI as age increases [3, 7, 47]. This can be attributed to the acquisition of immunity with age, resulting in some strains being cleared out. Other findings document an increased MOI with age, which could be a result of the protection of children under five due to the use of insecticide treated bed nets and other prevention approaches against mosquito bites that could make older children immune naïve [48]. We report an inverse correlation between MOI and age (for msp2) although the findings lack statistical significance. Other studies report a lack of correlation between MOI and age similar to the general findings from our study [28, 49].

The current study reports a lack of association between *P. falciparum* allelic families or MOI and clinical outcomes, particularly ACPR similar to studies done in Sudan, Uganda and Gabon [33, 50, 51]. Our findings are in contrast with those reported in Uganda where children infected with multiple strains were more likely to experience treatment failure than those infected with a single strain [22]. This conflict in results could be attributed to the differences in transmission and vector populations between the study areas. Further evidences such as meta-analyses are required to reach the conclusion on the role of msp polymorphisms on treatment outcomes among malaria patients.

Our findings suggest a lack of correlation between MOI and parasite density (at days 1 and 2). The findings are in match with other previous studies in Africa [52, 53] but in contrast with findings from other previous studies, which report an inverse correlation between parasite density and MOI [28]. An inverse correlation between haemoglobin values and MOI has been observed in our study. This finding is in contrast to a study done in Congo [53]. The reason for the inverse correlation between haemoglobin and MOI needs to be established. The high rate of antimalarial medication before clinical consultation could be the reason behind the observed lack of association between MOI and parasite density [54].

Findings from the present study serve as baseline data for future malaria epidemiological studies on malaria transmission at the study site and an evaluation of the influence of parasite genotypes on treatment outcomes among *P. falciparum* malaria patients.

## 5. Conclusions

Most malaria patients harbour polyclonal infections harbouring multiple genotypes and the high MOI displays the high genetic diversity of *P. falciparum* infection in the country. Inverse correlations between day 2 parasitaemia and ACPR and between haemoglobin values and MOI were reported in our study. No correlation was observed between parasite density or age and MOI. MOI did not influence ACPR among malaria patients. The observed high number of multiclonal isolates suggests a complex population structure of the parasite and may accelerate the spread of resistance over time.

## Figures and Tables

**Table 1:** *Plasmodium falciparum* merozoite surface protein 1 and 2 diversity.

Genes	Allele	Positive *n*/*N* (%)	Fragment sizes (bp)	Number of alleles	MOI
msp1	MAD20	51/102 (50)	150–300	08	1.63
	K1	77/102 (75.6)	160–300	09	1.50
	RO33	45/102 (43.6)	120–200	04	1.11
	MAD20/K1	31/100 (31.0)			
	MAD20/RO33	30/101(29.5)			
	K1/RO33	32.1/100 (32.1)			
	MAD20/K1/RO33	19/100 (19.0)			

msp2	3D7	94/115 (81.7)	100–400	15	1.58
	FC27	92/115 (76.9)	250–550	10	1.24
	3D7/FC27	75/115 (65.4)			

**Table 2:** Multiplicity of infection of msp1 and msp2 in malaria patients.

Parameters/variables	msp1	msp2	*p* values
Multiplicity of infection (MOI)	2.44	2.27	0.669
Monoclonal infection *n*/*N* (%)	42/102 (41.2%)	39/115 (39.9%)	0.456
Polyclonal infection	58/102 (58.8%)	69/115 (60.1%)	0.811
Overall mean multiplicity of infection	Mean MOI 2.36		NA

NA = not applied.

**Table 3:** Correlation between MOI and other variables.

Parameters/variables	Multiplicity of infection (MOI)			
	msp1		msp2	
	Correlation coefficient	*p* values	Correlation coefficient	*p* values
Age	0.074	0.521	−0.129	0.261
No of strains/bands	0.053	0.648	0.765	<0.001
Parasite density at day 1	0.133	0.244	0.043	0.712
Parasite density at day 2	0.219	0.054	−0.006	0.957
Haemoglobin	−0.223	0.049	−0.139	0.249

## Data Availability

The data used to support the findings are included within the article.

## Abbreviations

ACPR: Adequate clinical and parasitological response
ALU: Artemether-lumefantrine
COI: Complexity of infection
DBS: Dried blood spots
ETF: Early treatment failure
ITNs: Insecticide treated nets
LCF: Late clinical failure
LD: Linkage disequilibrium
LPF: Late parasitological failure
MRDT: Malaria rapid diagnostic test
MOI: Multiplicity of Infection
msp: Merozoite surface protein
WHO: World Health Organization.

## References

[1] World Health Organization (2022). World malaria report 2021.

[2] Miller L. H., Baruch D. I., Marsh K., Doumbo O. K. (2002). The pathogenic basis of malaria. *Nature*.

[3] Kateera F., Nsobya S. L., Tukwasibwe S. (2016). Malaria case clinical profiles and *Plasmodium falciparum* parasite genetic diversity: a cross sectional survey at two sites of different malaria transmission intensities in Rwanda. *Malaria Journal*.

[4] Eisen D., Billman-Jacobe H., Marshall V. F., Fryauff D., Coppel R. L. (1998). Temporal variation of the merozoite surface protein-2 gene of Plasmodium falciparum. *Infection and Immunity*.

[5] Dutta S., Lee S. Y., Batchelor A. H., Lanar D. E. (2007). Structural basis of antigenic escape of a malaria vaccine candidate. *Proceedings of the National Academy of Sciences*.

[6] Healer J., Murphy V., Hodder A. N. (2004). Allelic polymorphisms in apical membrane antigen-1 are responsible for evasion of antibody-mediated inhibition in *Plasmodium falciparum*. *Molecular Microbiology*.

[7] Usman-Yamman H., Omalu C. J. I., Abubakar A., Eke S., Eke S., Otuu C. A. (2021). Genetic diversity of plasmodium falciparum isolates in Minna, North Central Nigeria inferred by PCR genotyping of Merozoite surface protein 1 and 2. *Infection, Genetics and Evolution*.

[8] Zwetyenga J., Rogier C., Trape J. F. (1998). No influence of age on infection complexity and allelic distribution in *Plasmodium falciparum* infections in Ndiop, a Senegalese village with seasonal, mesoendemic malaria. *The American Journal of Tropical Medicine and Hygiene*.

[9] Holder A., Blackman M. (1994). What is the function of MSP-I on the malaria merozoite?. *Parasitology Today*.

[10] File T., Golassa L., Dinka H. (2022). Plasmodium falciparum clinical isolates reveal analogous circulation of 3D7 and FC27 allelic variants and multiplicity of infection in urban and rural settings: the case of adama and its surroundings, oromia, Ethiopia. *Journal of Parasitology Research*.

[11] Abdel Hamid M., Mohammed S. B., El Hassan I. M. (2013). Genetic diversity of Plasmodium falciparum field isolates in Central Sudan inferred by PCR genotyping of merozoite surface protein 1 and 2. *North American Journal of Medical Sciences*.

[12] Snounou G., Singh B. (2002). Nested PCR analysis of Plasmodium parasites. *Malaria Methods and Protocols*.

[13] Duah N. O., Matrevi S. A., Quashie N. B., Abuaku B., Koram K. A. (2016). Genetic diversity of Plasmodium falciparum isolates from uncomplicated malaria cases in Ghana over a decade. *Parasites & Vectors*.

[14] Chen Q., Schlichtherle M., Wahlgren M. (2000). Molecular aspects of severe malaria. *Clinical Microbiology Reviews*.

[15] Pacheco M. A., Lopez-Perez M., Vallejo A. F., Herrera S., Arévalo-Herrera M., Escalante A. A. (2016). Multiplicity of infection and disease severity in Plasmodium vivax. *PLoS Neglected Tropical Diseases*.

[16] Vafa M., Troye-Blomberg M., Anchang J., Garcia A., Migot-Nabias F. (2008). Multiplicity of Plasmodium falciparum infection in asymptomatic children in Senegal: relation to transmission, age and erythrocyte variants. *Malaria Journal*.

[17] A-Elbasit I. E., ElGhazali G., A-Elgadir T. M. E. (2007). Allelic polymorphism of MSP2 gene in severe P. falciparum malaria in an area of low and seasonal transmission. *Parasitology Research*.

[18] Kidima W., Nkwengulila G. (2015). Plasmodium falciparum msp2 genotypes and multiplicity of infections among children under five years with uncomplicated malaria in Kibaha, Tanzania. *Journal of parasitology research*.

[19] Nderu D., Kimani F., Karanja E. (2019). Genetic diversity and population structure of Plasmodium falciparum in kenyan–ugandan border areas. *Tropical Medicine and International Health*.

[20] Anderson T. J. C., Haubold B., Williams J. T. (2000). Microsatellite markers reveal a spectrum of population structures in the malaria parasite Plasmodium falciparum. *Molecular Biology and Evolution*.

[21] Lê H. G., Kang J.-M., Jun H. (2019). Changing pattern of the genetic diversities of Plasmodium falciparum merozoite surface protein-1 and merozoite surface protein-2 in Myanmar isolates. *Malaria Journal*.

[22] Kyabayinze D. J., Karamagi C., Kiggundu M. (2008). Multiplicity of Plasmodium falciparum infection predicts antimalarial treatment outcome in Ugandan children. *African Health Sciences*.

[23] Marwa K. J., Konje E. T., Kapesa A., Kamugisha E., Mwita S., Swedberg G. (2021). Artemether–lumefantrine and dihydroartemisinin–piperaquine treatment outcomes among children infected with uncomplicated Plasmodium falciparum malaria in Mwanza, Tanzania. *Tropical Medicine and Health*.

[24] World Health Organization (2010). *Basic Malaria Microscopy: Part I. Learner’s Guide*.

[25] Somé A. F., Bazié T., Zongo I. (2018). Plasmodium falciparum msp 1 and msp 2 genetic diversity and allele frequencies in parasites isolated from symptomatic malaria patients in Bobo-Dioulasso, Burkina Faso. *Parasites & Vectors*.

[26] World Health Organization (2008). *Methods and Techniques for Clinical Trials on Antimalarial Drug Efficacy: Genotyping to Identify Parasite Populations: Informal Consultation Organized by the Medicines for Malaria Venture and Cosponsored by the World Health Organization*.

[27] World Health Organization (2009). *Methods for Surveillance of Antimalarial Drug Efficacy*.

[28] Mohammed H., Assefa A., Chernet M., Wuletaw Y., Commons R. J. (2021). Genetic polymorphisms of Plasmodium falciparum isolates from Melka-Werer, North East Ethiopia based on the merozoite surface protein-2 (msp-2) gene as a molecular marker. *Malaria Journal*.

[29] Mita T., Jombart T. (2015). Patterns and dynamics of genetic diversity in Plasmodium falciparum: what past human migrations tell us about malaria. *Parasitology International*.

[30] Ferreira M. U., da Silva Nunes M., Wunderlich G. (2004). Antigenic diversity and immune evasion by malaria parasites. *Clinical and Vaccine Immunology*.

[31] Durand P., Tibayrenc M., Renaud F. (2003). Significant linkage disequilibrium and high genetic diversity in a population of Plasmodium falciparum from an area (Republic of the Congo) highly endemic for malaria. *The American Journal of Tropical Medicine and Hygiene*.

[32] Congpuong K., Sukaram R., Prompan Y., Dornae A. (2014). Genetic diversity of the msp-1, msp-2, and glurp genes of P lasmodium falciparum isolates along the Thai-Myanmar borders. *Asian Pacific Journal of Tropical Biomedicine*.

[33] Mahdi Abdel Hamid M., Elamin A. F., Albsheer M. M. A. (2016). Multiplicity of infection and genetic diversity of Plasmodium falciparum isolates from patients with uncomplicated and severe malaria in Gezira State, Sudan. *Parasites & Vectors*.

[34] Soe T. N., Wu Y., Tun M. W. (2017). Genetic diversity of Plasmodium falciparum populations in southeast and western Myanmar. *Parasites & Vectors*.

[35] Ghanchi N. K., Mårtensson A., Ursing J. (2010). Genetic diversity among Plasmodium falciparum field isolates in Pakistan measured with PCR genotyping of the merozoite surface protein 1 and 2. *Malaria Journal*.

[36] Chenet S. M., Branch O. H., Escalante A. A., Lucas C. M., Bacon D. J. (2008). Genetic diversity of vaccine candidate antigens in Plasmodium falciparum isolates from the Amazon basin of Peru. *Malaria Journal*.

[37] Kumkhaek C., Phra-Ek K., Rénia L. (2005). Are extensive T cell epitope polymorphisms in the Plasmodium falciparum circumsporozoite antigen, a leading sporozoite vaccine candidate, selected by immune pressure?. *The Journal of Immunology*.

[38] Oboh M. M., Ndiaye T., Diongue K. (2020). Is genetic diversity of plasmodium falciparum suggestive of malaria endemic zone in West Africa? An attempt to respond by a cross-sectional comparative study between Senegal and Nigeria. *medRxiv*.

[39] Ndiaye T., Sy M., Gaye A., Ndiaye D. (2019). Genetic polymorphism of merozoite surface protein 1 (msp1) and 2 (msp2) genes and multiplicity of Plasmodium falciparum infection across various endemic areas in Senegal. *African Health Sciences*.

[40] Read A. F., Taylor L. H. (2001). The ecology of genetically diverse infections. *Science*.

[41] Tusting L. S., Bousema T., Smith D. L., Drakeley C. (2014). Measuring changes in *Plasmodium falciparum* transmission: precision, accuracy and costs of metrics. *Advances in Parasitology*.

[42] De Roode J. C., Pansini R., Cheesman S. J. (2005). Virulence and competitive ability in genetically diverse malaria infections. *Proceedings of the National Academy of Sciences*.

[43] Sherman I. W. (1998). *Malaria: Parasite Biology, Pathogenesis and Protection*.

[44] Kiwanuka G. N., Joshi H., Isharaza W. K., Eschrich K. (2009). Dynamics of *Plasmodium falciparum* alleles in children with normal haemoglobin and with sickle cell trait in western Uganda. *Transactions of the Royal Society of Tropical Medicine and Hygiene*.

[45] Mwingira F., Nkwengulila G., Schoepflin S. (2011). Plasmodium falciparum msp1, msp2 and glurp allele frequency and diversity in sub-Saharan Africa. *Malaria Journal*.

[46] Magesa S. M. (1999). Malaria parasite dynamics: epidemiology, allelic diversity and turnover rates of Plasmodium falciparum infections in Tanzanian children.

[47] Agyeman-Budu A., Brown C., Adjei G. (2013). Trends in multiplicity of Plasmodium falciparum infections among asymptomatic residents in the middle belt of Ghana. *Malaria Journal*.

[48] Pemberton-Ross P., Smith T. A., Hodel E. M., Kay K., Penny M. A. (2015). Age-shifting in malaria incidence as a result of induced immunological deficit: a simulation study. *Malaria Journal*.

[49] Atroosh W. M., Al-Mekhlafi H. M., Mahdy M. A., Saif-Ali R., Al-Mekhlafi A. M., Surin J. (2011). Genetic diversity of Plasmodium falciparum isolates from Pahang, Malaysia based on MSP-1 and MSP-2 genes. *Parasites & Vectors*.

[50] Kiwuwa M. S., Ribacke U., Moll K. (2013). Genetic diversity of Plasmodium falciparum infections in mild and severe malaria of children from Kampala, Uganda. *Parasitology Research*.

[51] Bouyou-Akotet M. K., M’Bondoukwé N. P., Mawili-Mboumba D. P. (2015). Genetic polymorphism of merozoite surface protein-1 in Plasmodium falciparum isolates from patients with mild to severe malaria in Libreville, Gabon. *Parasite*.

[52] Mohammed H., Kassa M., Mekete K., Assefa A., Taye G., Commons R. J. (2018). Genetic diversity of the msp-1, msp-2, and glurp genes of Plasmodium falciparum isolates in Northwest Ethiopia. *Malaria Journal*.

[53] Gueye N. S. G., Ntoumi F., Vouvoungui C. (2018). Plasmodium falciparum merozoite protein-1 genetic diversity and multiplicity of infection in isolates from Congolese children consulting in a pediatric hospital in Brazzaville. *Acta Tropica*.

[54] Marwa K. J., Liwa A. C., Konje E. T., Mwita S., Kamugisha E., Swedberg G. (2022). Lumefantrine plasma concentrations in uncontrolled conditions among patients treated with artemether-lumefantrine for uncomplicated plasmodium falciparum malaria in Mwanza, Tanzania. *International Journal of Infectious Diseases*.

